# Pain…. from analgesic care to decennal experience

**DOI:** 10.1186/1824-7288-41-S1-A10

**Published:** 2015-09-24

**Authors:** Maria(Gabriella) Di Ventura, Laura Wlderk, Assunta Fabi, Gustavo Caoci, Luisa Pieragostini

**Affiliations:** 1 Unità Operativa Complessa Neonatal Hospital San Filippo Neri Rome, Italy

## 

Our little Neonatal Intensive and Pathology care centre (T.I.N.) with “preterm infants not many but very important ones” welcomed pathological and premature infants. In Italian born around 500/540 thousand children a year, 7.5% of these infants are born before 37 weeks Gestational Age (G.A.) and 2% with very low birth weight and severe prematurity. The mechanical ventilation, the parenteral feeding, the diagnostic/therapeutic methods and the maternal milk have increased the survival of these small babies. Today we know that the baby, even if extremely premature, feels pain and that perception is stronger if G.A. at birth is lower. Therefore in our T.I.N., the professional care is addressed to humanization of clinical treatments, very important part of “right to life, but above all of “right to the best quality of life”. From warm and comfortablemother's womb to T.I.N.environment, premature infants experiencesan unknown world made of lights, sounds, smells and knowpain, solitude, the contact with different hands, jeopardizing the normal maturation process of his brain function. Therefore, in our T.I.N., since the nineties visual and acoustic stimuli have been minimized giving great importance to postural control, the basic element of the harmonious balance and proper movement, with the help of little nests inside the incubator in order to protect and content the baby looking forward to the “kangaroo mother care”. Later in 2004, we integrated the protocol of care with and without analgesia and pharmacological (sensory saturation). In the last decade, in our department there were technical improvements that formed the group of newborn pain with operators always updated and representatives in each round. Infants were treated following the directions of the Group of Study of Pain of Italian Society of Neonatology implementing, especially, the use of pain scales; in fact, a good pain management can not ignore the above.

In everyday life has become part of our team a physical therapist specializing in Italian Association of Infant Massage and a psychologist. Also important is the presence of representatives of “breastfeeding” to inform and encourage the mothers of the little ones about the importance of breast milk. Last but not least the opening day of the T.I.N. parents.
